# Factors Affecting Herd Status for Bovine Tuberculosis in Dairy Cattle in Northern Thailand

**DOI:** 10.1155/2017/2964389

**Published:** 2017-05-03

**Authors:** Tawatchai Singhla, Sukolrat Boonyayatra, Veerasak Punyapornwithaya, Kimberly L. VanderWaal, Julio Alvarez, Srinand Sreevatsan, Somphorn Phornwisetsirikun, Jamnong Sankwan, Mongkol Srijun, Scott J. Wells

**Affiliations:** ^1^Department of Food Animal Clinic, Faculty of Veterinary Medicine, Chiang Mai University, Chiang Mai, Thailand; ^2^Graduate School, Chiang Mai University, Chiang Mai, Thailand; ^3^Veterinary Public Health Center for Asia Pacific, Faculty of Veterinary Medicine, Chiang Mai University, Chiang Mai, Thailand; ^4^Department of Veterinary Population Medicine, College of Veterinary Medicine, University of Minnesota, St. Paul, MN, USA; ^5^Chiang Mai Provincial Livestock Office, Chiang Mai, Thailand; ^6^Chiang Rai Provincial Livestock Office, Chiang Rai, Thailand; ^7^Center for Animal Health and Food Safety, University of Minnesota, St. Paul, MN, USA

## Abstract

The objective of this case-control study was to identify farm-level risk factors associated with bovine tuberculosis (bTB) in dairy cows in northern Thailand. Spatial analysis was performed to identify geographical clustering of case-farms located in Chiang Mai and Chiang Rai provinces in northern Thailand. To identify management factors affecting bTB status, a matched case-control study was conducted with 20 case-farms and 38 control-farms. Case-farms were dairy farms with at least single intradermal tuberculin test- (SIT-) reactor(s) in the farms during 2011 to 2015. Control-farms were dairy farms with no SIT-reactors in the same period and located within 5 km from case-farms. Questionnaires were administered for data collection with questions based on epidemiological plausibility and characteristics of the local livestock industry. Data were analyzed using multiple logistic regressions. A significant geographic cluster was identified only in Chiang Mai province (*p* < 0.05). The risk factor associated with presence of SIT-reactors in dairy herds located in this region was purchasing dairy cows from dealers (OR = 5.85, 95% CI = 1.66–20.58, and *p* = 0.006). From this study, it was concluded that geographic clustering was identified for dairy farms with SIT-reactors in these provinces, and the cattle movements through cattle dealers increased the risks for SIT-reactor farm status.

## 1. Introduction

Bovine tuberculosis (bTB), caused by* Mycobacterium bovis*, is an important disease affecting the agricultural economy of Thailand and is associated with public health risks as a zoonotic disease [1]. The disease affects cattle health and well-being, decreases milk production in dairy cows, and has negative impacts on profitability and trade [2, 3]. Data on bTB prevalence in developing countries is minimal, and the limited information available may not represent the true epidemiological status of the disease as a result of lack of eradication and control programs [4].

In Thailand, the Department of Livestock Development (DLD) is implementing a program to test and eradicate infected dairy cattle annually based on application of the* caudal fold* SIT test to all adult dairy cows (age ≥ 1 year). SIT-reactors in infected dairy herds are culled and slaughtered [5]. However, active surveillance at slaughter houses has not been implemented in Thailand. This control program has been effective in reducing bTB prevalence in dairy herds, but it has not eradicated the disease. In northern Thailand, especially in Chiang Mai province, where the largest population of dairy cattle in the region is located, bTB in dairy farms has been reported almost every year. In 2012, the prevalence of bovine tuberculosis in this region was estimated at 0.30% at the animal level and 4.38% at the herd level [5]. Ongoing transmission of bTB in northern Thailand, despite the implementation of a test-and-slaughter program for many years, indicates the need to identify risk factors for bTB in dairy herds. Understanding these factors will assist in creation of an effective control plan for bTB in this region.

Several studies have identified risk factors for bTB across multiple countries worldwide [6, 7]. Many studies identified infected herds using results from both SIT testing and postmortem examination [8–10], while other studies utilized results only from SIT testing to identify bTB status of cattle herds [6, 11, 12]. In Thailand, data on necropsy findings are usually not available in most cases. Therefore, bTB status of a cattle herd in Thailand is usually based on the results of SIT testing.

Risk factors affecting bTB can be divided into 3 levels, including animal, farm, and regional or country levels [13]. In infected herds, older animals are more likely to be exposed to* M. bovis *than younger ones [11]. Large dairy herds are more likely to have infected animals compared to small herds [11, 13]. Moreover, increasing herd size may increase probability of a false positive reactor, given limitations in the specificity of SIT tests [14]. Animal purchase or movement of dairy cows has also been associated with the prevalence of the disease [3, 4, 8, 9, 12]. Other risk factors appear to vary across regions and husbandry practices [3, 15]. In regions where dairy cows are not confined in barns and have access to natural areas, such as in Europe, Africa, and parts of North America, risk factors include contact with wildlife as reservoirs of the disease [1, 10, 13]. However, wildlife-cattle interactions are very rare in Thailand. Risk factors at animal and herd levels have not been extensively evaluated in Thailand, and this epidemiological information can help to develop an effective control plan for bTB in the region. The current study was conducted to identify herd-level risk factors associated with bovine tuberculosis in dairy cows in Chiang Mai and Chiang Rai provinces, Thailand.

## 2. Materials and Methods

### 2.1. Study Design and Study Setting

**Figure 1:**
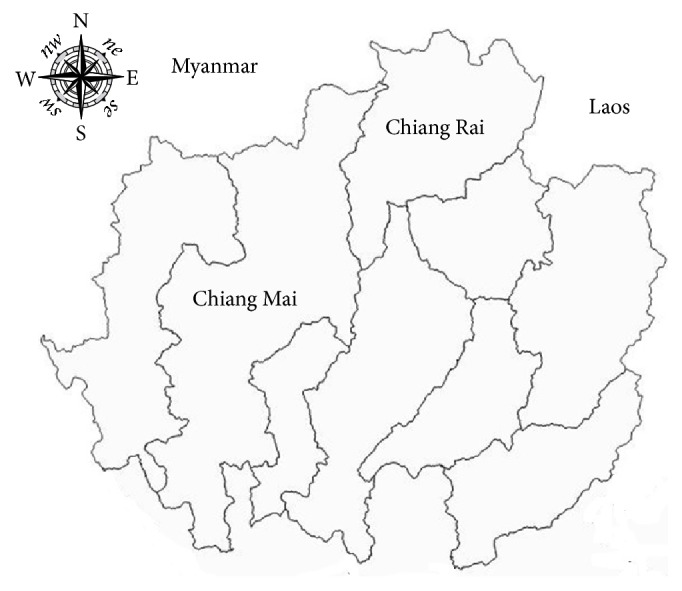
Geographical location of Chiang Mai and Chiang Rai provinces in northern Thailand.

This study was a retrospective case-control study conducted in Chiang Mai and Chiang Rai provinces in Thailand. Chiang Mai and Chiang Rai provinces are located in northern Thailand adjacent to Myanmar and Laos (Figure 1). The number of dairy cattle in Chiang Mai and Chiang Rai provinces is approximately 60% of the total population of dairy cattle in the northern region of Thailand [16].

### 2.2. Case and Control Selection

Bovine tuberculosis test status information from dairy herds in Chiang Mai and Chiang Rai provinces during 2011 to 2015 was obtained from the Thailand DLD. Case-farms were defined as dairy farms with history of at least one* caudal fold* SIT-reactor during 2011 to 2015. Control-farms were selected from the population of dairy farms in these provinces with no detected or reported SIT-reactor during the same period. An increased skinfold thickness ≥ 5 mm after 72 h at the injection site of intradermal injection of purified protein derivative of* M. bovis* was the cut-off used to categorize* caudal fold* SIT-reactors [17].

### 2.3. Data Collection

Geographical coordinates of a total of 931 farms in the selection provinces were obtained from the DLD for spatial cluster analysis. Regarding the definitions of case-farms described in previous section, 16 case- (SIT-positive) and 844 SIT-negative farms in Chiang Mai province and 4 case- (SIT-positive) and 67 SIT-negative farms in Chiang Rai province were included for spatial cluster analysis.

A matched case-control study was designed to identify farm management factors associated with bTB in Chiang Mai and Chiang Rai provinces. Each case-farm was geographically matched with 2 control-farms located within a 5 km radius of the case-farm. In Chiang Rai province, 1 of the case-farms did not have any neighboring dairy farm located within 5 km. Therefore, 20 case- and 38 control-farms were included in this study. Questionnaires were administered to case- and control-farms to collect farm-level information on general herd management, herd profile, breed, history of bTB and cattle movements in and out of the herd, disease outbreaks, other animal species kept, types and levels of herd contacts, water sources, and workers information through interviews of the owners of case- and control-farms by a study investigator. The questionnaire consisted of closed- and open-ended questions and was pretested on 5 farms in Chiang Mai province to assess the clarity of questions, with modification to capture aspects of management initially missed.

### 2.4. Statistical Analysis

Spatial clustering analysis of 20 case-farms and 911 SIT-negative farms was conducted using the Bernoulli model of the spatial scan statistic. The null hypothesis was that the distribution of case-farms locations was random. The model compares the observed number of case-farms within all possible circular spatial windows in the study area with the expected number of bTB-positive farms. The test was performed by using the Satscan software version 9.1.1.

For identification of farm-level risk factors, data were analyzed in 2 stages using R version 3.2.2. In the first stage, categorical and continuous variables were first screened using univariate logistic regressions with a random effect representing each case-control grouping. Evaluation of multicollinearity among predictor variables was assessed using chi-square test for categorical variables (*p* < 0.05) and Pearson product-moment correlation for continuous variables (cor ≥ 0.5). In case of multicollinearity (i.e., *p* < 0.05 or cor ≥ 0.5), the variable with higher biological plausibility was retained for multivariate analysis.

In the second stage of risk factor analyses, variables from the univariate analysis with* p* ≤ 0.2 and without marked multicollinearity among the candidate variables were included in the full multivariate logistic regression for model selection. A stepwise procedure based on the Akaike Information Criterion (AIC) was performed by using the* bestglm* package [18] in R version 3.2.2. The model with the lowest AIC value was selected as the final model. If several candidate models had similarly low AIC value (difference in AIC value < 2), the principal of parsimony was used and the model with the fewest parameters was preferred as the final model. The final model's goodness-of-fit was evaluated on the basis of the Hosmer–Lemeshow statistic. The ability of the model to discriminate between cases and controls, or model sensitivity, was tested using a Receiver Operating Characteristic (ROC) and area under the curve (AUC). Models with an AUC value greater than 0.8 or between 0.7 and 0.8 were considered to have good moderate discriminative capacities, respectively. Accuracy of the model prediction was assessed using cross-validation method with random sampling of observed data for model prediction. The results from the model prediction were compared to observed data and the outcome was shown based on percentage of accuracy.

## 3. Results

### 3.1. Descriptive Data

All dairy farms in the case-control study (data available from questionnaire) were small-holders, with a mean herd size of 21 milking cows (range: 4–47). Mean herd sizes of dairy farms in Chiang Mai and Chiang Rai provinces were 21 and 24 milking cows, respectively. Most of the farms housed cattle in free stalls with zero grazing. Tied stall housing was observed in 20% of farms in Chiang Mai province, but not in Chiang Rai province. In all study herds, cows typically were fed twice daily with concentrate feed and ad libitum roughage. Roughage and concentrate feed were fed separately; total mixed ration feeding systems were not used in this area. Farms typically utilized a bucket-type milking machine for milk collection.

### 3.2. Spatial Analysis

**Figure 2:**
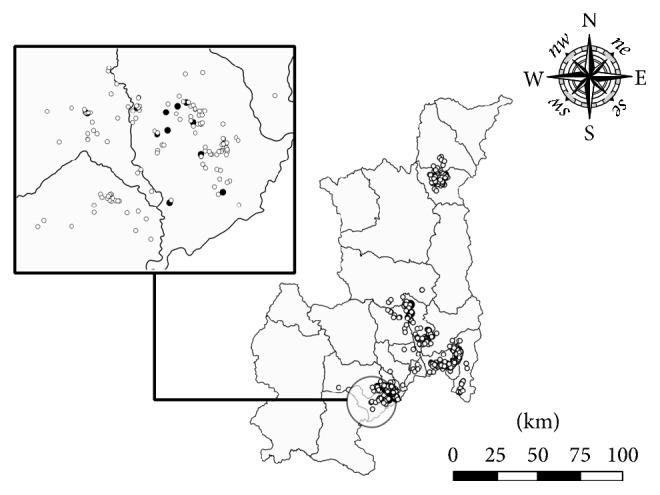
Locations of case- and control-farms in Chiang Mai province during 2011–2015. Circle indicates cluster of infected farms. Black dots indicate case-farms and white dots indicate control-farms.

**Figure 3:**
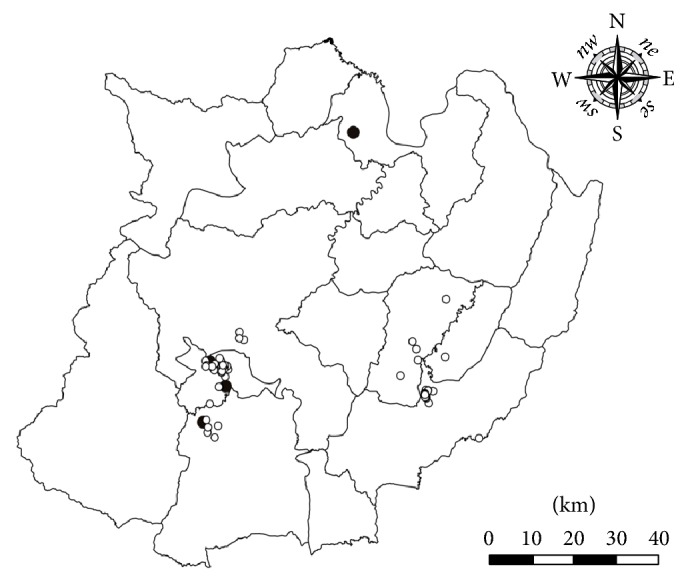
Locations of case- and control-farms in Chiang Rai province during 2011–2015. Black dots indicate case-farms and white dots indicate control-farms.

A cluster of case-farms was identified in 4 districts of Chiang Mai province with 14.7 km in radius (*p* < 0.05) as shown in Figure 2. A total of 107 farms (11.5%) consisting of 12 case-farms and 95 SIT-negative farms were included in the significant cluster. In contrast, no significant cluster of case-farms was identified in Chiang Rai province. In Chiang Rai province, 4 case-farms were located in 3 different districts with distance of approximately 10–100 km apart (Figure 3).

### 3.3. Univariate Analysis for the Matched Case-Control Study

Eight variables were identified from univariable analysis, with* p* ≤ 0.2, including purchasing cows from dealers, purchasing cows from central Thailand, imported cows presented in the farm, number of open heifers, introducing cows >1 time/year, deworming of dry cows, selling cows to farms within the same cooperative, and selling 1-2 cows per time (Table 1). No multicollinearity among variables was observed, so these variables were offered to the multivariate logistic regression model process.

### 3.4. Multivariate Analysis for the Matched Case-Control Study

The primary factor associated with bTB status in dairy herds identified from the final multiple logistic regression model was purchasing cows from dealers (OR = 5.85; 95% CI = 1.66–20.58; *p* = 0.006; Table 2). As a subanalysis, considering farms located within the significant cluster in Chiang Mai province identified from spatial analysis, 9 out of 12 case-farms (75%) in this cluster purchased cows from dealers, whereas 29% of the control-farms in this cluster purchased cows from dealers.

The final model for bTB risk factors prediction fitted the data well when tested with the Hosmer–Lemeshow goodness-of-fit *p* > 0.05. The area under the ROC curve (AUC) calculated for this model was 0.74 (95% CI: 0.7–0.8); therefore, the final model had moderate discrimination capacity. The accuracy of the model prediction was 74% when calculated with cross-validation.

## 4. Discussion

From cluster analysis of spatial data of SIT-reactor and nonreactor farms in this study, significant clustering was identified in Chiang Mai province. Identification of spatial clusters of disease and causal mechanisms related to this clustering could be useful for improving eradication programs in this area. The primary risk factor for SIT-reactivity at farm-level was related to cattle movement. In the final logistic regression model of the current study, purchasing cows from cattle dealers posed the highest risk for bTB infection, and case-farms had odds of engaging in this practice nearly 6 times higher than control-farms. This practice poses risk to cattle producers because the farm of origin of the cattle purchased is not known by the new owners, including whether the animals had originated from farms with history of SIT-reactor cattle. Cattle movements have also been identified as major factors associated with bTB outbreaks in other regions, including cattle moved from endemic areas to previously bTB-free farms [8, 12, 19].

In this study, a large cattle market was located within the identified bTB geographic cluster. The convenience of cattle trading due to the accessibility to the cattle market within the clustered area may have led to an increased rate of purchasing cows through dealers, which consequently increased frequencies of cattle movements in this area. In Thailand, while cattle movements across province boundaries are regulated by the Thailand DLD, with creation of movement records as a result, movements within a province are not regulated. Therefore, farmers purchasing cattle from unknown sources within a province (through cattle dealers in this area) may have experienced higher disease introduction risks associated with these unregulated animal movements compared to other farmers.

A limitation of this study was that the prevalence of bTB in Chiang Mai and Chiang Rai was quite low, with limited power for clustering analysis. Therefore, further spatial analyses should be performed in other areas in this region to evaluate the consistency of these findings across the overall region to improve understanding of the epidemiology of the disease.

Risk factors associated with bTB status have been previously reported in other regions of the world, including herd size, use of specific management practices, lack of performance of diagnostic tests, and movement of cattle [13]. The movement of cattle has been shown to be a major risk for bTB infection when cattle are moved from an endemic area to a bTB-free area [13]. Information regarding epidemiologic information including risk factors and geographic clustering of bTB in Thailand is minimal. This study identifying an important role of cattle movements as risk provides a starting point for understanding the epidemiology of bTB in northern Thailand, as the characteristics and management practices of these farms were generally similar to other dairy cattle farms in the region, with some variation in sources of roughage or milking procedures.

Eradiation of bTB with endemic in cattle populations around the world is challenging. One recent report has estimated the costs in a developing country under different scenarios of cattle testing [20] demonstrating, for example, that testing higher proportions of cattle and using a severe test interpretation are ultimately more cost-effective at the population level than alternatives. However, another important factor to be considered in eradication programs is the risk of disease movements facilitated through animal movements, especially in situations involving use of imperfect tests as the case for bTB in Thailand and many other regions of the world. Improved understanding of the risks associated with cattle movements allows the potential for targeted testing of high-risk populations such as cattle moving through dealers to help mitigate this risk to support disease eradication programs.

## 5. Conclusions

From results of this study, we conclude that purchasing dairy cows from cattle dealers was strongly associated with SIT-reactivity on dairy farms in northern Thailand. A prudent disease control recommendation is for cattle owners to decrease the risk of bTB infection through cattle introductions and to ask for verification that dairy cattle introduced to their herds are from bTB-test negative farms. Additional study to provide further clarification of these risk factors would lead to improved understanding of the epidemiology of bTB infection on dairy farms in this region, which could support the likelihood of successful bTB eradication and control programs in the country.

## Figures and Tables

**Table 1:** Univariate analysis of variables as *p* ≤ 0.2 considered for multivariate analysis and the percentage of the distribution of the risk factors in case- and control-farms.

Variable	Case (%)	Control (%)	*p *value
Purchasing cows from dealers	75.0	36.8	0.046
Purchasing cows from central Thailand	50.0	23.7	0.046
Imported cows presented in the farm	65.0	39.5	0.065
Number of open heifers	—	—	0.114
Introducing cows > 1 time/year	50.0	28.9	0.116
Deworming of dry cows	80.0	60.5	0.140
Selling cows to farms in the same cooperative	35.0	18.4	0.166
Selling 1-2 cows per time	80.0	92.1	0.192

**Table 2:** Results of multiple logistic regression analysis.

Variables	Coefficient	SE	adj. OR	95% CI	*p* value
Purchasing cows from dealers	1.52	0.66	5.85	1.66–20.58	0.006
